# Bovine respiratory syncytial virus seroprevalence and risk factors in non-vaccinated dairy cattle herds in Brazil

**DOI:** 10.1186/s12917-018-1535-8

**Published:** 2018-06-27

**Authors:** Ingrid Bortolin Affonso Lux Hoppe, Andréa Souza Ramos de Medeiros, Clarice Weis Arns, Samir Issa Samara

**Affiliations:** 1 Faculdade de Ciências Agrárias e Veterinárias (FCAV), Departamento de Medicina Veterinária Preventiva e Reprodução Animal, Univ Estadual Paulista – UNESP, Via de Acesso Prof. Paulo Donato Castellane, km 05, Jaboticabal, São Paulo CEP: 14.884-900 Brazil; 2 Instituto de Biologia, Universidade Estadual de Campinas – UNICAMP, Cidade Universitária, Caixa-postal: 6109, Campinas, São Paulo CEP: 13.083-970 Brazil

**Keywords:** Bovine respiratory syncytial virus, Seroprevalence, Risk factors, Dairy cattle herds

## Abstract

**Background:**

The cattle industry is one of the most important Brazilian agribusiness sectors and is a strong contributor to the national economy. Annually about 44.6 million calves are bred, which makes the optimal management of these animals extremely important. Several diseases can affect the initial stages of the bovine production chain, being the bovine respiratory syncytial virus (BRSV) one of the most relevant pathogens. This study aimed to characterize the epidemiology of BRSV infection in dairy cattle herds of São Paulo State, Brazil, using serological and risk factors analyses. For that, 1243 blood samples were collected of animals from 26 farms and a questionnaire about possible risk factors for BRSV prevalence was performed. The obtained blood sera were analyzed using virus neutralization test (VNT).

**Results:**

VNT results showed high BRSV prevalence in dairy cattle herds, reaching 79.5% of seropositivity. The BRSV seroprevalence among studied farms ranged from 40 to 100%. The analysis of risk factors indicated that the age group and the occurrence of coinfection with bovine herpesvirus 1 (BoHV-1) and bovine viral diarrhea virus 1 (BVDV-1) should be associated with a higher prevalence of BRSV, while natural suckling was considered a protective factor.

**Conclusions:**

The study showed that adult animals over 1 year old are an important risk factor for the high seroprevalence of BRSV in herds. The high BRSV prevalence associated with BoHV-1 and BVDV-1 suggests that biosecurity measures should be applied in order to reduce viral dissemination. Additionally, the natural suckling may be an important management to protect calves from high BRSV seroprevalence.

## Background

Bovine respiratory syncytial virus (BRSV) is an economically significant pathogen in cattle production [1], as it is one of the most important causes of lower respiratory tract infections in calves [2]. In dairy cattle, BRSV infection usually occurs in young calves aged between 2 weeks and 9 months [3]. Adult animals with subclinical infection are the main source of infection, since reinfections are common in the herds [1, 4, 5].

BRSV, bovine herpesvirus 1 (BoHV-1), bovine viral diarrhea virus (BVDV) and bovine parainfluenza type-3 (PI-3) are considered primary agents involved in the bovine respiratory complex. Additionally, secondary infection by *Pasteurella multocida*, *Histophilus somni* and mycoplasmas contribute to the aggravation of the disease [6]. Clinical signs are characterized by respiratory symptoms, initially with moderated intensity, such as nasal and ocular discharges which can be aggravated leading to pneumonia. However, mainly in calves, an acute and severe onset is also observed, due to maternal antibodies not effectively protect against BRSV infection [3].

Considering the high prevalence of the disease, several studies determined risk factors involved in the epidemiology of BRSV. In Europe, risk factors were mainly attributed to herd size, herd density, purchasing of new animals, geographic location of the farms, herd type and concomitant BVDV infection [7–11]. Similar studies have also been performed in some Latin American countries and they showed that most of the animals probably have already been exposed to the virus with consequent high BRSV prevalence in cattle herds. In these countries, herd size, age group, presence of bordering farms, herd type and geographic location of the farms were the main risk factors associated with BRSV infection [12–16].

In Brazil, BRSV was first diagnosed in calves in the state of Rio Grande do Sul [17] and some studies have shown that BRSV infection is widespread in Southern and Southeastern Brazil, with high serological prevalence rates [18–20]. Nevertheless, research has not been conducted in order to verify possible risk factors involved in BRSV epidemiology. Due to this, the current study aimed to determine antibody prevalence against BRSV and investigate some risk factors associated with BRSV seroprevalence in herds of an important milk producing region in São Paulo State, Brazil.

## Methods

### Study area and sample collection

The study was performed on 26 dairy cattle herds in 12 municipalities in the Northern region of the São Paulo State, Southeastern Brazil. This region produces about 10 million liters of milk annually [21]. The evaluated herds had between 6 and 150 animals and no animals were vaccinated against the pathogens associated with respiratory diseases. The farms were located in a region classified as Aw by the climatic classification of Koeppen, characterized by dry winter with average temperature higher than 18 °C in the coldest month and precipitation below 60 mm in the driest month. The altitude of these areas was 440 to 617 m above sea level [22].

Sampling of each farm was calculated [23] with an expected BRSV prevalence of 80% [19] with acceptable error of 5% and confidence level of 95%. After setting the number of samples, bovines of all categories and age group were randomly selected. According to the management adopted in the herds, the age group was defined as ≤12 months old “calf” and > 12 months old “adult”. In some farms, the number of samples collected was higher than that suggested by the mathematical calculation, once it was also used for serodiagnosis of BoHV-1 and BVDV-1. On farms with a small herd size, up to 30 animals, all of them were sampled.

The samples from 1243 animals were collected from April 2012 to June 2012 as shown in Table 1. Blood samples were collected by jugular or coccygeal vein puncture using disposable needles and vacuum tubes. Samples remained at room temperature for 1 h for coagulation and after being transported refrigerated to the laboratory, they were centrifuged at 1080×*g* for 10 min to obtain the sera. The sera were aliquoted in 1.5 mL identified microtubes that were stored in freezer at − 20 °C until analyses.

**Table 1:** Total of blood samples collected in 26 dairy cattle herds in the Northern region of the São Paulo State, Southeastern Brazil

Dairy cattle herds	Total number of animals	Number of blood samples		Total of samples
		Adult	Calf	
1	75	47	25	72
2	38	21	14	35
3	88	50	23	73
4	55	31	20	51
5	50	25	19	44
6	120	41	25	66
7	7	5	2	7
8	9	9	0	9
9	120	85	16	101
10	150	81	37	118
11	36	24	12	36
12	120	83	36	119
13	80	59	18	77
14	40	28	3	31
15	40	26	9	35
16	15	10	5	15
17	26	20	6	26
18	15	12	3	15
19	60	37	13	50
20	100	61	27	88
21	16	11	5	16
22	6	6	0	6
23	52	37	11	48
24	22	19	3	22
25	62	44	17	61
26	22	19	3	22
**Total**		**891**	**352**	**1243**

### Virus neutralization test

The presence of antibodies to BRSV was tested by virus neutralization test (VNT). Serum samples were thawed, inactivated in water bath at 56 °C for 30 min, and diluted in duplicates from 1:2 to 1:1024 in 96-well microplates with 50 μL of 200 TCID_50_ BRSV suspended in Eagle’s minimum essential medium (DIFCO E-MEM®). The viral strain was previously titrated [24]. Following incubation at 37 °C in 5% CO_2_ atmosphere for 1 h, 50 μL of Madin & Darby Bovine Kidney (MDBK) cells suspension, in E-MEM and 10% bovine fetal serum solution were added to the wells. Then, the plates were re-incubated in similar conditions for 96 h. Each test included a back titration and cell culture control. Samples were positive when cytopathic effect was inhibited at 1:2 dilution, and the titration was expressed as the inverse of the dilution, as geometric mean.

### Risk factors

During the sample collection, a questionnaire was administered to the owner or manager of each farm with the purpose of identifying potential risk factors related to epidemiology of BRSV. Thus, the questionnaire was designed according to data of risk factors described in the literature [8, 10, 15, 16] in addition to the information of the management adopted in dairy farms of São Paulo State. The variables explored were: herd size (≤75, > 75 animals), herd density (≤5, > 5 animals/hectare), age group (≤12 months old “calf”, > 12 months old “adult”), breed (Holstein, mixed), type of reproduction (natural mating, artificial insemination), purchase of animals (last 6 months: yes, no), cleaning of facilities (often, rarely), historical of respiratory disease (last 6 months: yes, no), quarantine (yes, no), abortions (yes, no), BoHV-1 infection (serodiagnosis: yes, no), BVDV-1 infection (serodiagnosis: yes, no), type of calves housing (individual, collective), type of calves feeding (natural suckling, artificial), presence of others domestic animals (yes, no), presence of wild ruminant animals (yes, no).

The variables “herd size” and “herd density” were based on the average data; “age group” was defined from the management adopted on the farms; “quarantine” refers to the isolation of purchased animals before adding them to the herd. The serodiagnosis of BoHV-1 and BVDV-1 was performed at the same time as BRSV (unpublished data). The variables “disinfection of the umbilical cord” and “colostrum feeding” were not analyzed because these practices were applied in all dairy farms visited. Additionally, the data related to climatic classification and altitude were not included in the analysis because they were similar in all 26 farms.

### Statistical analysis

The chi-square tests were employed to compare the BRSV, BoHV-1 and BVDV-1 seropositivity and also with “age group”. Fisher’s exact test was used to compare BRSV status according to the expected prevalence of 80% (≥80%, “high”; < 80%, “low”) and the other variables. Only the variables with *p* < 0,2 (two-tailed Fisher) were analyzed by a logistic regression model. The analyses were performed using the Epi Info™ program v. 7.0.

## Results

Serum samples from 1243 animals belonging to 26 dairy cattle herds were taken and tested by VNT, in which 988 (79.5%) were seropositive to BRSV. Regarding the age group, 87% (767/ 891) of the serum samples were positives for adult animals while the prevalence rate in calves was 62.8% (352/221). Antibodies to BRSV were detected in all cattle herds, with prevalence rates ranging from 40 to 100%. The mean antibody titers for adult animals was 2 to 512, and for calves, 2 to 32. Therefore, prevalence of BRSV both in herds and animals was considered high.

The chi-square test showed association of BRSV seropositivity with “age group” and animals tested seropositives to BoHV-1 and BVDV-1 (Table 2). Nevertheless, the Fisher’s exact test only detected statistical difference with the variable “type of calves feeding” (Table 3). In this case, the relative risk (RR) value was less than one, i.e., the factor “natural suckling” was considered protective. Logistic regression (values of *p* < 0.2, two-tailed Fisher) did not show significant results, suggesting that the variables analyzed were not risk factors for the high seroprevalence of BRSV in the studied population.

**Table 2:** Risk factors associated with BRSV in 1243 animals from 26 dairy cattle herds in São Paulo State, Brazil

Factor		BRSV		Relative risk (RR)	Confidence interval (CI) (95%)		*p* value
		Positive	Negative		Limit inferior	Limit superior	
Age group	> 12 months (adult)	775	116	1.44	1.32	1.57	< 0.0001
	≤12 months (calf)	213	139				
BoHV-1	Seropositive	565	85	1.22	1.15	1.29	< 0.0001
	Seronegative	423	170				
BVDV-1	Seropositive	298	33	1.19	1.13	1.25	< 0.0001
	Seronegative	690	222

**Table 3:** Risk factors associated with high and low bovine respiratory syncytial virus prevalence at herd level in 26 dairy cattle herds in São Paulo State, Brazil

Factor	Condition	BRSV status		RR	CI (95%)		*p* value
		High	Low		Limit inferior	Limit Superior	
Herd size (> 75 animals)	Yes	3	2	1.40	0.59	3.34	0.422
	No	9	12				
Herd density (> 5 animals/ha)	Yes	5	4	1.34	0.60	3.05	0.387
	No	7	10				
Breed (Holstein)	Yes	7	7	1.20	0.51	2.81	0.488
	No	5	7				
Type of reproduction (natural mating)	Yes	2	8	0.32	0.09	1.17	0.420
	No	10	6				
Purchasing of animals (last six months)	Yes	6	10	0.63	0.28	1.41	0.237
	No	6	4				
Cleaning of facilities (rarely)	Yes	7	9	0.88	0.38	2.01	0.536
	No	5	5				
Historical of respiratory disease (last six months)	Yes	7	6	1.40	0.60	3.28	0.347
	No	5	8				
Quarantine (absence)	Yes	10	11	1.19	0.37	3.81	0.578
	No	2	3				
Abortions	Yes	5	6	0.97	0.42	2.26	0.632
	No	7	8				
Type of calves housing (collective)	Yes	9	14	0.39	0.24	0.65	0.085
	No	3	0				
Type of calves feeding (natural suckling)	Yes	3	10	0.33	0.12	0.96	0.023
	No	9	4				
Presence of others domestic animals	Yes	11	13	0.92	0.21	3.92	0.720
	No	1	1				
Presence of wild ruminant animals	Yes	5	9	0.61	0.26	1.43	0.224
	No	7	5				

## Discussion

This is the first epidemiological study to assess risk factors for BRSV seroprevalence carried out in Brazil. Even though BRSV prevalence of 79.5% in the animals sampled was similar to that estimated, the prevalence in adult animals was higher than that expected, reaching 87% of samples. In calves, the seroprevalence was lower than that found in adult animals (62.8%) and could be even lower once VNT does not allow the distinction between antibodies from colostrum and natural infection. Thus, this study demonstrated that the prevalence of BRSV antibodies was higher in adult animals, as previously reported in other countries [13, 16].

Adult animals are associated with high seroprevalence of BRSV as consequence of a repeated exposure to the virus infection throughout their life and possibility of reinfections. Similarly, the highest antibody titers were associated with non-vaccinated adult cattle, probably due to the exposure to successive viral reinfections, which results in a booster effect on antibody titers [25]. Other factor related to high antibody titers is recent BRSV infections, which can be confirmed only by paired serology, antibody screening in calves after the period of colostral antibody detection or viral detection by direct methods. As respiratory disease was not reported in half of the herds studied, it is indicative that BRSV infection can be subclinical. This is consistent with previous reports [2]. Herds can remain free of clinical BRSV infection for many years even in areas of high prevalence of the virus [26].

The presence of other pathogens is also associated with the prevalence of BRSV [8, 11, 14, 16]. This information explains the association of BRSV serological prevalence with the prevalences of BoHV-1 and BVDV-1. The infection by these viral agents is also reported in Brazilian herds, with high prevalences [27, 28]. BVDV infection can cause impairment of the animal’s immune function and thereby decrease resistance to other infections [8]. The synergistic effects of BVDV with other respiratory pathogens have been observed [29, 30]. Thus, health status of the herds may also be affected indirectly by BVDV control measures [8].

Dairy cattle herds in São Paulo State usually have poor biosecurity measures, such as the lack of quarantine of newly purchased animals, lack of diagnosis of respiratory diseases (particularly for BRSV) and vaccination is rarely performed against these viruses. Therefore, we hypothesized that risk factors for the seroprevalence of BoHV-1, BVDV-1 and BRSV in the studied population likely to overlap.

Despite the logistic regression not confirming “type of calves feeding” variable as a risk factor for high prevalence of BRSV, the Fisher’s exact test detected “natural suckling” as a protective factor. “Natural suckling” would be important as it may be able to reduce the risk of calves becoming infected by BRSV. Weaning can be stressful and results in impaired immune function, which may further exacerbate a BRSV exposure. Suckling reduces the occurrence of diarrhea, prevents the abnormal behavior of cross-suckling of other calves and improves animal health [31, 32]. Prior to the current study there have been no report about “natural suckling” and its relationship with BRSV seroprevalence or its role as a protective factor, therefore, based on the results presented, it has the potential to decrease seroprevalence to BRSV.

Similarities were observed among the results found at the present study and those previously obtained by others conducted in Brazil [18–20]. In Latin America countries, equivalents prevalences of BRSV have also been reported [12, 14–16], as well as difficulties in detecting the risk factors involved in the dissemination of the agent, even using different forms of sampling and analyzing a considerable number of variables. Thus, the dynamics of infection may differ even in a particular country or geographic area [26].

The high serological prevalence of BRSV found in this study shows the importance to know more about this infection since it is not considered important in the country, mainly due to the lack of diagnosis. The awareness of the risk factors involved in the BRSV dissemination can allow understanding its mechanisms, even though, as in other studies, these factors were not very clear. Thereby, further studies as a complement to the current one should be performed until concrete information has been found.

## Conclusions

Adult animals over 1 year old can present high seroprevalences of BRSV and are an important risk factor for the virus maintenance in herds. Additionally, the concomitant seroprevalence of BoHV-1 and BVDV-1 leads us to suggest that biosecurity measures to reduce viral dissemination could be applied. Non-stressful management, like keeping calves with their mothers promoting natural suckling, may constitute a practical management strategy to reduce the seroprevalence of BRSV in Brazilian dairy herds.

## Abbreviations

BoHV-1: Bovine herpesvirus type-1
BRSV: Bovine respiratory syncytial virus
BVDV: Bovine viral diarrhea virus
CI: Confidence interval
MDBK: Madin & Darby Bovine Kidney
PI-3: Bovine parainfluenza type-3
RR: Relative risk
VNT: virus neutralization test
